# Transcriptomic and Quantitative Proteomic Analyses Provide Insights Into the Phagocytic Killing of Hemocytes in the Oyster *Crassostrea gigas*

**DOI:** 10.3389/fimmu.2018.01280

**Published:** 2018-06-11

**Authors:** Shuai Jiang, Limei Qiu, Lingling Wang, Zhihao Jia, Zhao Lv, Mengqiang Wang, Conghui Liu, Jiachao Xu, Linsheng Song

**Affiliations:** ^1^Key Laboratory of Experimental Marine Biology, Institute of Oceanology, Chinese Academy of Sciences, Qingdao, China; ^2^Liaoning Key Laboratory of Marine Animal Immunology and Disease Control, Dalian Ocean University, Dalian, China; ^3^University of Chinese Academy of Sciences, Beijing, China

**Keywords:** *Crassostrea gigas*, transcriptome, quantitative proteome, phagocyte, oxidative killing, lysosome, cathepsin L

## Abstract

As invertebrates lack an adaptive immune system, they depend to a large extent on their innate immune system to recognize and clear invading pathogens. Although phagocytes play pivotal roles in invertebrate innate immunity, the molecular mechanisms underlying this killing remain unclear. Cells of this type from the Pacific oyster *Crassostrea gigas* were classified efficiently in this study *via* fluorescence-activated cell sorting (FACS) based on their phagocytosis of FITC-labeled latex beads. Transcriptomic and quantitative proteomic analyses revealed a series of differentially expressed genes (DEGs) and proteins present in phagocytes; of the 352 significantly high expressed proteins identified here within the phagocyte proteome, 262 corresponding genes were similarly high expressed in the transcriptome, while 140 of 205 significantly low expressed proteins within the proteome were transcriptionally low expressed. A pathway crosstalk network analysis of these significantly high expressed proteins revealed that phagocytes were highly activated in a number of antimicrobial-related biological processes, including oxidation–reduction and lysosomal proteolysis processes. A number of DEGs, including oxidase, lysosomal protease, and immune receptors, were also validated in this study using quantitative PCR, while seven lysosomal cysteine proteases, referred to as cathepsin Ls, were significantly high expressed in phagocytes. Results show that the expression level of cathepsin L protein in phagocytes [mean fluorescence intensity (MFI): 327 ± 51] was significantly higher (*p* < 0.01) than that in non-phagocytic hemocytes (MFI: 83 ± 26), while the cathepsin L protein was colocalized with the phagocytosed *Vibrio splendidus* in oyster hemocytes during this process. The results of this study collectively suggest that oyster phagocytes possess both potent oxidative killing and microbial disintegration capacities; these findings provide important insights into hemocyte phagocytic killing as a component of *C. gigas* innate immunity.

## Introduction

The process of phagocytosis has been shown to play essential roles in pathogen defense and immune surveillance (1, 2). Several kinds of leukocytes in vertebrates (especially mammals) undertake phagocytic killing activities as part of innate immunity (3): neutrophils, for example, are one kind of phagocyte that disarm and kill microbial pathogens either intracellularly as part of phagocytosis or extracellularly *via* antimicrobial peptides (4). Previous work has shown that both dendritic cells (DCs) and macrophages phagocytose and enclose bacteria in membrane-bound phagosomes (preferentially phagolysosomes) before killing these invading pathogens *via* the activity of reactive oxygen species (ROS) or lysosomal hydrolases (5, 6). DCs act as dominant antigen-presenting cells, processing foreign substances from pathogens and presenting them to T cells to initiate adaptive immunity (7). Research on lower vertebrates such as teleosts has shown that B lymphocytes possess potent phagocytic and bactericidal capabilities, providing important insights into the evolution of phagocytes (8). Although invertebrates lack the T-cell receptors and major histocompatibility complex molecules necessary for mediated adaptive immunity, long-lived species are thought to be able to cope with dynamically changing immune challenges *via* innate immunity (9, 10). An increasing body of evidence suggests that phagocytes play important roles in invertebrate innate immune responses (11): in the Pacific white shrimp *Litopenaeus vannamei*, for example, hyalinocytes and granulocytes both phagocytose fungi and bacteria as ROS production increases (12), while phagocytes in the Hawaiian bobtail squid *Euprymna scolopes* degrade engulfed bacteria *via* the lysosomal proteolytic pathway (13). Previous research has also shown a significant reduction in hemocyte phagocytosis in the blue mussel *Mytilus edulis* following cadmium exposure; this finding highlights the regulation of hemocyte phagocytic activity in innate immunity (14). A number of immune-related genes involved in hemocyte phagocytosis in *M. edulis* were also significantly upregulated following *Vibrio splendidus* challenge (15).

The molecular mechanisms that underlie phagocytic killing within immune responses have been studied extensively in mammals (16–18). Previous work has shown that mutual recognition between membrane receptors and pathogens leads to receptor clustering and membrane protrusions, which eventually enables microbial engulfment (19). Free radicals, including ROS, also exert potent antimicrobial activity against engulfed microbes across a broad spectrum of host immune responses (20). Internalized microbes are generally trapped within phagosomes that undergo further fusion with lysosomes which have potent microbicidal abilities as a result of their highly acidic pH (between 4.5 and 5.0) and proteolytic activities (21, 22). Cathepsin, a lysosomal proteolytic protease, is one of the most important proteases owing to its strong bacterial killing and decomposition activity (23). Cathepsin L in mouse macrophages exhibits strong bactericidal activity toward *Mycobacterium tuberculosis* (24), while cathepsin G exerts antibacterial activity against *Pseudomonas aeruginosa* in human neutrophils (25). Although the mechanisms that underlie phagocytic killing abilities have been well documented in mammals, such mechanisms in invertebrates remain poorly understood.

The Pacific oyster, *Crassostrea gigas*, one of the most important aquaculture species worldwide, is a keystone taxon in coastal and estuarine ecology (26). As oysters are sessile marine invertebrates that inhabit estuarine and intertidal regions, they are subjected to extraordinary abundant microbial challenges from the surrounding environment (27). The elucidation of phagocytic killing is, therefore, necessary to understand the molecular mechanisms that underlie innate immune defense in *C. gigas*. In this study, phagocytes from this species that engulfed FITC-labeled latex beads were classified using fluorescence-activated cell sorting (FACS). Differentially expressed genes (DEGs) and proteins (DEPs) within these phagocytes, identified *via* transcriptomic and quantitative proteomic analyses, were compared with those in non-phagocytic hemocytes. A crosstalk network was constructed to illustrate the hyperactive immune pathways involved in hemocyte phagocytosis. A number of significant DEPs associated with these pathways were validated by qPCR at the messenger RNA (mRNA) level, and the high expression of lysosomal protease cathepsin L in phagocytes was determined using specific antibodies. Moreover, the potential role of cathepsin L in immune defense against microbial infection was further investigated in light of its colocalization with engulfed pathogens during *C. gigas* hemocyte phagocytosis by confocal microscopy.

## Materials and Methods

### Animal Rearing and Manipulation

Pacific oyster (*C. gigas*) specimens with lengths between 10 and 15 cm and weights between 150 and 200 g were collected from a local breeding farm in Tsingtao, China. These specimens were acclimated in aerated and filtered seawater at 18°C, and fed with spirulina powder every other day for 2 weeks prior to use in experiments. Seawater in the aquaria was replaced every day, and all experiments were performed following local and central government regulations. All experiments involving animals reported in this study were approved by the Ethics Committee of the Institute of Oceanology, Chinese Academy of Sciences (28).

### Hemocyte Preparation

Approximately 1 mL of hemolymph per oyster was extracted from the pericardial cavity of adult *C. gigas* specimens using a syringe and needle (0.9 by 25 mm) after the shell had been carefully opened. The hemolymph was immediately mixed with pre-chilled anticoagulant acid citrate dextrose solution (ACD-A; 7.3 g/L citric acid, 22.0 g/L sodium citrate, and 24.5 g/L dextrose) at a 7:1 volume/volume ratio, pooled into sterilized 50 mL Falcon tubes, pelleted at 800 × *g* at 4°C for 10 min, and washed twice with modified Leibovitz L15 medium (supplemented with 0.54 g/L KCl, 0.6 g/L CaCl_2_, 1 g/L MgSO_4_, 3.9 g/L MgCl_2_, 20.2 g/L NaCl, 100 U/mL penicillin G, 40 µg/L gentamycin, 100 µg/mL streptomycin, 0.1 µg/mL amphotericin B, and 10% fetal bovine serum). Hemocytes were resuspended in modified Leibovitz L15 medium (29) and stored on ice for 30 min to reduce spontaneous aggregation.

### FACS Analysis of Phagocytic and Non-Phagocytic Hemocytes

Phagocytic and non-phagocytic hemocytes from 600 oysters were sorted in triplicate using FACS. In each case, 200 oysters were randomly grouped for hemocyte preparation and cell sorting, as previously described (30). Hemocytes were collected and incubated with FITC-labeled latex beads (2 µm) at a ratio of 1:100 (hemocytes/beads) and rotated continuously (30 rpm) for 3 h at 18°C. Trypan blue (1.2 mg/mL) was used to quench surface-bound FITC-labeled beads; then, hemocytes were washed twice with modified Leibovitz L15 medium and analyzed using a FACS Aria II flow cytometer (Becton Dickinson Biosciences). FITC-positive and -negative cells were gated using a FL1 channel and sorted based on their fluorescence intensity. Flow cytometry data were analyzed using a BD FACS Diva system (Becton Dickinson Biosciences) and the sorted phagocytes and non-phagocytic hemocytes were further validated using flow cytometry and fluorescence microscopy.

### Library Preparation and RNA Sequencing

Total RNA was isolated from sorted phagocytes and non-phagocytic hemocytes using Trizol reagent (Invitrogen) following the manufacturer’s instructions. RNA molecules greater than 200 nucleotides (nt) in length were purified and their concentration was measured using a Nanodrop 2000 spectrophotometer (Thermo Scientific). Integrity in each case was checked using an Agilent 2100 Bioanalyzer (Agilent Technologies) and ribosomal RNA was removed using a RiboMinus Eukaryote Kit for RNA-Seq (Invitrogen). A single-end fragment library was constructed using a SOLiD Total RNA-Seq Kit (Life Technologies), while ribo-minus RNA was fragmented using RNase III and purified using the RiboMinus Concentration Module (Invitrogen). Fragments of RNA were linked with adaptors using a hybridization master mix (SOLiD Total RNA-Seq Kit), and reverse transcription was performed. Purified complementary DNA (cDNA) was selected based on size following DNA electrophoresis with a Novex TBE-Urea Gel (Invitrogen) at 180 V for 20 min. A gel block containing cDNA lengths between 150 and 250 nt was precisely excised and used as an amplification template. All PCR reactions were performed at 95°C for 5 min before being thermally cycled 15 times at 95°C for 30 s, 62°C for 30 s, and 72°C for 30 s. All the components used in this amplification were from a SOLiD Total RNA-Seq Kit and the yield and size distribution of PCR products was analyzed using an Agilent 2100 Bioanalyzer. Emulsion PCR and bead enrichment were performed using a SOLiD EZ BeadTM system (Life Technologies). A workflow analysis was carried out to verify the quality and density of template beads, and about 120 million enriched beads for each sample were deposited on a sequencing slide. Libraries were sequenced using the SOLiD 4 platform and color-space reads were generated. Three biological transcriptomic sequencing replicates were performed in each case, and all raw data were deposited in the NCBI Sequence Read Archive database under the accession number SRP133110.

### Transcriptome Bioinformatic Analysis

Read alignment was performed with the software BioScope (Life Technologies) using the *C. gigas* genome as the reference.^1^ The expression level of each gene was estimated using the frequency of clean reads in the corresponding sample, and the RPKM method was applied to calculate read density. Gene expression values are summarized in Table S1 in Supplementary Material, and the false discovery rate (FDR) was used to determine *p* value thresholds in multiple tests. Absolute values of log_2_ > 1 and FDR ≤ 0.001 were set as thresholds to determine DEGs between phagocytes and non-phagocytic hemocytes. DEG annotations were performed by running this assembly against the genome of *C. gigas* (Table S2 in Supplementary Material). All blast results were imported into the software Blast2GO to map sequences onto gene ontology (GO) (Table S3 in Supplementary Material). The Kyoto Encyclopedia of Genes and Genomes Ortholog database (KEGG^2^) was applied using the software BLASTx (E < 10^−5^) to analyze metabolic pathways in *C. gigas* and the results from the KEGG analysis are presented in Table S4 in Supplementary Material.

### Protein Extraction, Digestion, and iTRAQ Labeling

All sorted phagocytic and non-phagocytic hemocytes were lysed in a buffer (7 mol/L urea, 2 mol/L thiourea, 4% NP40, 20 mmol/L Tris–HCl, pH 8.0) and centrifuged at 4°C for 15 min at 30,000 × *g*. The resultant supernatant was mixed with dithiothreitol at a final concentration of 10 mmol/L and incubated at 56°C in the dark for 1 h to reduce the disulfide bonds. Iodoacetamide (IAM; 55 mM) was added and the solution was incubated for 1 h in the dark. Proteins were precipitated using acetone and dissolved in 500 µL of 0.5 M triethylammonium bicarbonate (TEAB, Applied Biosystems) followed by the addition of Trypsin Gold (Promega) at a 30:1 ratio of protein to trypsin and incubated at 37°C for 16 h. Tryptic peptides from phagocytic and non-phagocytic hemocytes were reconstituted in 0.5 M TEAB and iTRAQ labeling was performed with 100 µg of protein samples according to the manufacturer’s protocol, using an 8 × plex iTRAQ reagent (Applied Biosystems). Tags 114 and 117 were used to label the peptides from phagocytic and non-phagocytic hemocytes, respectively.

### Strong Cation Exchange (SCX) Chromatography Assay

Strong Cation Exchange chromatography was carried out using an LC-20AB HPLC Pump system (Shimadzu). In this protocol, iTRAQ-labeled peptides were reconstituted using 4 mL of buffer A (25 mM NaH_2_PO_4_ in 25% acetonitrile, pH 2.7) and loaded onto an Ultremex SCX column (Phenomenex). Peptides were eluted with buffer A for 10 min before a linear gradient of 5–60% buffer B (25 mmol/L NaH_2_PO_4_, 1 mol/L KCl in 25% acetonitrile, pH 2.7) elution for 27 min, followed by another linear gradient of 60–100% buffer B for 1 min. Eluted peptides were pooled into 20 fractions and desalted using a Strata X C18 column (Phenomenex).

### LC-ESI-MS/MS Analysis

Each peptide fraction was vacuum-dried and re-suspended in buffer I (5% acetonitrile, 0.1% formic acid) before adjusting the final concentration for each sample to approximately 0.5 µg/µL. The supernatant (10 µL) was loaded onto a C18 trap column equipped with an LC-20AD nanoHPLC (Shimadzu). Eluted peptides were further separated using an analytical form of this column with a linear gradient of 2–35% buffer II (95% acetonitrile, 0.1% formic acid) at a flow rate of 300 nL/min for 35 min, followed by a linear gradient of buffer II of 35–60% for 5 min, which was increased to 80% for 2 min. This buffer concentration was maintained at 80% for 4 min and then reduced to 5% for 1 min. LC-ESI-MS/MS analysis was performed using a Triple TOF 5600 system (AB SCIEX) fitted with a Nanospray III source (AB SCIEX) using a pulled quartz tip as the emitter (New Objectives). The resolving power of this equipment was greater than, or equal to, 30,000 full widths at half maximum (FWHM). Data were acquired using an ion spray voltage of 2.5 kV, a curtain gas at 30 psi, and a nebulizer gas at 15 psi. A series of survey scans were acquired in 250 ms to enable an information-dependent acquisition model. A total of 30 product ion scans were collected, which exceeded a threshold of 120 counts per second incorporating a charge-state of +2 to +5 for each cycle. The total cycle time was fixed at 3.3 s and the Q2 transmission window was set at 100 Da for 100%. Four time bins were summed for each scan at a pulse frequency value of 11 kHz by monitoring the 40 GHz multichannel TDC detector using four-anode channels, while a sweeping collision energy setting of 35 ± 5 eV coupled with iTRAQ to adjust the rolling collision energy was applied to all precursor ions for collision-induced dissociation. Dynamic exclusion was set for half peak width (15 s) and the precursor was refreshed from the exclusion list. All raw mass spectrometry data were deposited in the Integrated Proteome Resources (iProX^3^) database under the accession number IPX0001165000.

### Bioinformatic Analysis of the Proteome

Raw data files acquired from the Orbitrap were converted into MGF format, and protein identification was carried out using the Mascot search engine (Matrix Science) against the *C. gigas* genome database. The mass tolerance of precursor and fragment ions during data calibration was also determined using the same software, taking into account one missed cleavage in trypsin digests. Thus, Gln -> pyro-Glu (N-term Q), oxidation (M), and deamidated (NQ) were set as potential variable modifications, while carbamidomethyl (C), iTRAQ 8-plex (N-term), and iTRAQ 8-plex (K) were used as fixed modifications. Peptide charge states were set at +2 and +3, and an automatic decoy database search was performed using the Mascot software by selecting the decoy checkbox in which a random database sequence was generated and tested for raw spectra in addition to the actual version. To reduce the false peptide identification probability, proteins determined in this step were required to contain at least one unique peptide with significance scores greater than, or equal to 20 at a 99% confidence level based on Mascot probability analysis. Identified proteins presented in Table S5 in Supplementary Material were required to contain two, or more, unique peptides for quantification, and their ratios were weighted and normalized using the Mascot median approach. Thus, protein ratios of *p* < 0.05 and more than a 1.2-fold change were considered significant in this analysis. Annotation of DEPs was performed by running the assembly against the *C. gigas* genome, as well as the COG and KEGG databases using BLASTx (E < 10^−5^). All blast results were imported into the software Blast2GO to map sequences onto GO terms, while the KEGG database was used to analyze *C. gigas* metabolic pathways. The bioinformatic DEP analysis for phagocytes generated in this study is summarized in Table S6 in Supplementary Material.

### Protein Network Mapping

**Figure 1:**
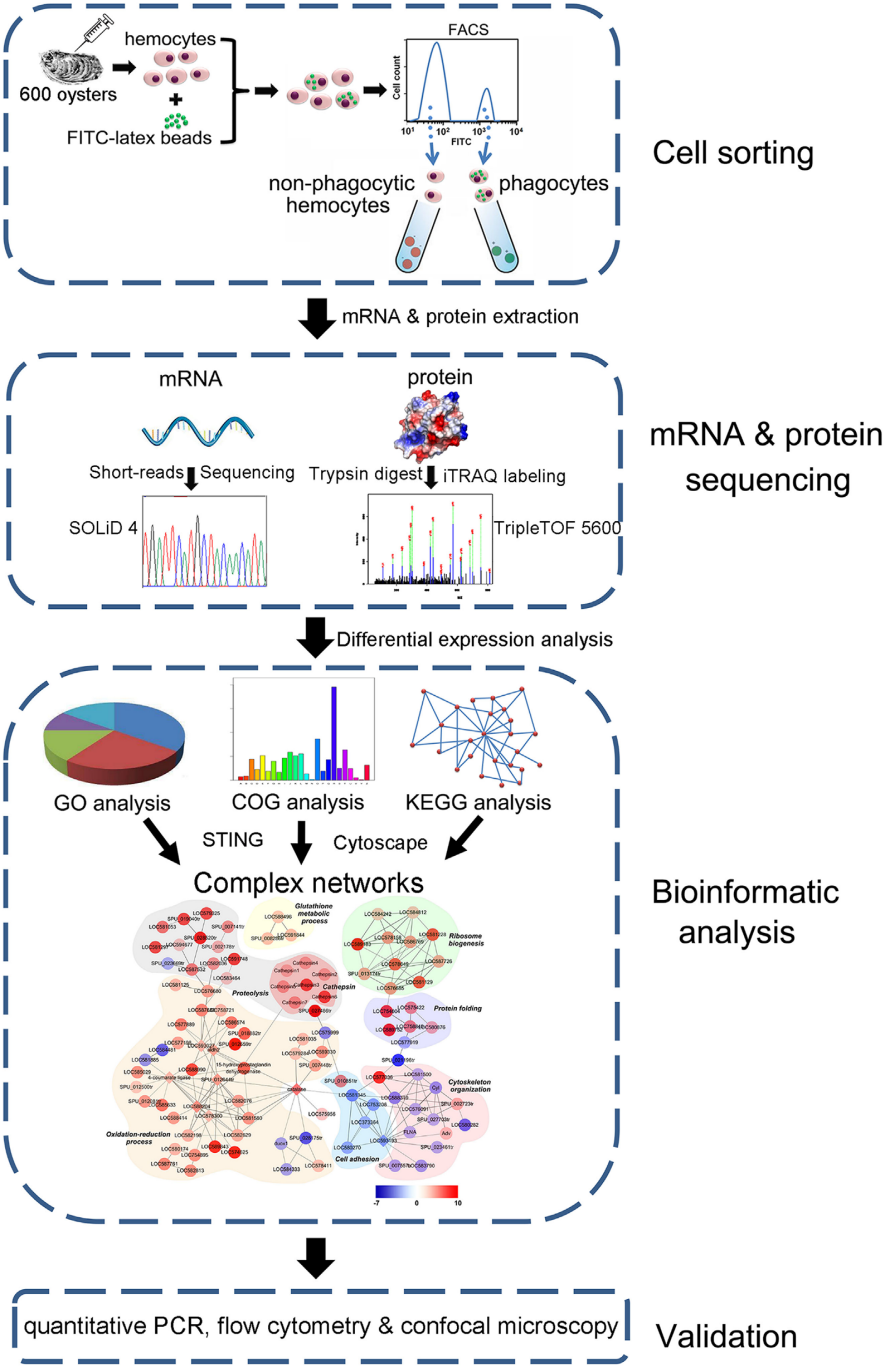
Pipeline overview of transcriptome and proteome analysis of *Crassostrea gigas* phagocytes. The hemocytes are collected, incubated with FITC-labeled latex beads, and sorted using FACS to prepare both phagocytes and non-phagocytic hemocytes. Differential expression in phagocytes is revealed using both transcriptome and quantitative proteome analyses. A complex network is constructed based on differentially expressed proteins and further validated using qPCR, flow cytometry, and confocal microscopy.

A protein interaction map was generated in this analysis as previously described (31). Proteins differentially expressed (*p* < 0.05) between phagocytic and non-phagocytic hemocytes were used to generate a network with the STRING software, while interactions were established, manually adjusted, and visualized using the Cytoscape software with an organic layout. Nodes were colored on the basis of maximum log_2_ ratio and placed into different functional groups. A pipeline overview of complex network construction based on DEPs is presented in Figure 1.

### qPCR Gene Expression Analysis

Phagocytic and non-phagocytic hemocyte cDNAs were prepared as described above. The mRNA expression profiles of 13 DEGs were determined using SYBR Green fluorescent qPCR with the oyster reference gene encoding elongation factor 1 as the internal control (32). This SYBR Green qPCR assay was performed using an ABI PRISM 7500 Sequence Detection System (Applied Biosystems) following the manufacturer’s protocols, and relative gene expression levels were analyzed using the 2^−ΔΔCT^ method (33). This experiment was performed three times and all data are presented in terms of relative gene expression (*N* = 6). Primer nucleotide sequences are summarized in Table 1.

**Table 1:** Primer nucleotide sequences used in this study.

Primer	Sequence (5′–3′)
**Clone primer**	
T3 primer	AATTAACCCTCACTAAAGGG
Oligo(dT)-adaptor	GGCCACGCGTCGACTAGTAC(T)17
Oligo(dG)-adaptor	GGCCACGCGTCGACTAGTAC(G)10
T7 promoter primer	TAATACGACTCACTATAGGG
T7 terminator primer	TGCTAGTTATTGCTCAGCGG
Cathepsin L forward	ATGTTGCGCGTTGCTGTAG
Cathepsin L reverse	TTACACAGTTGGGTAACTGGCA
**Recombination primers**	
Cathepsin L forward	GGAATTCCATATGTTGCGCGTTGCTGTAG
Cathepsin L reverse	CCGCTCGAGCACAGTTGGGTAACTGGCA
**RT primers**	
EF forward	AGTCACCAAGGCTGCACAGAAAG
EF reverse	TCCGACGTATTTCTTTGCGATGT
C1ql4 forward	AGCGGGGTCTATGTTTTCCAAT
C1ql4 reverse	CTTTGTTAGGCGAAGAACGACC
Scavenger receptor forward	CGGCAAGCACAGAGGACAAC
Scavenger receptor reverse	TCGTAATCAAAGGCAGTGGGTG
Cathepsin L forward	ACACCTACAGACTGGGGATGAAC
Cathepsin L reverse	CAGTCAACTGAGTCCGGTAGGTCT
Proline amino peptidase forward	GAGTGCTTACTACAGGAGGCTTACC
Proline amino peptidase reverse	GCTTTTTTCAACATCTCATCATCAA
Amino oxidase forward	GGGCAACGACCGAGTCCTCT
Amino oxidase reverse	GGGCAACGACCGAGTCCTCT
Dipeptidase forward	ACATTCCAAGGATCCGACAGG
Dipeptidase reverse	GTATCCCCTGAGAAGTGGTGACA
Macrophage mannose receptor forward	CAAAAGTCTCCCACACTCTCCCT
Macrophage mannose receptor reverse	GGAAAATAGGGTCGTCAATAGGAAT
Fascin forward	CGGGAATTACTTGGGAAACAG
Fascin reverse	CCTCCGTACATTGGATCTCGTC
Formin forward	CTGGTCGTAGATGTGAATGCG
Formin reverse	AGAGGTAACTTGACTAAAGCTCCTGT
Ubiquitin E3 ligase forward	CGAGGCGCACCCAATAAGT
Ubiquitin E3 ligase reverse	CTGCCAATACATTCCAGTTCC
Integrin β forward	TGTCCTTACTATGCTGGGCTGAT
Integrin β reverse	CTGGTGGGTACGACATTGCT
α-crystallin forward	CACAGTGAAGACCGTGGACC
α-crystallin reverse	TTTATCGGGAAGAAGGTAGGAC
Galectin 2 forward	GCTCTGGACAAGACGGCTGG
Galectin 2 reverse	GAAATGTTGTCAGGATTCCCACG

### Cloning and Sequencing the Cathepsin L cDNA

Hemocyte total RNA was extracted as described above, genomic DNA was digested using RNase-free DNase (Promega), and first-strand cDNA was synthesized using M-MLV reverse transcriptase (Promega). The cathepsin L cDNA was amplified using ExTaq DNA polymerase (Takara) and PCR amplification in this case was performed over 32 cycles at 95°C for 5 min, at 54°C for 30 s, and at 72°C for 1 min. The PCR product was gel-purified, cloned into a pMD18-T simple vector (Takara), and verified by sequencing. The cathepsin L cDNA was further amplified using primers with endonuclease sites, which are summarized in Table 1.

### Prokaryote Expression and Purification of Recombinant Proteins

The PCR product of the cathepsin L cDNA was gel-purified and cloned into a pET-30a expression vector (Novagen) that included a His-tag at the 3′ end. This recombinant plasmid was transformed into *Escherichia coli* DH5α competent cells, and clones with inserts in the forward orientation were screened using PCR and further confirmed by nucleotide sequencing. The valid recombinant plasmid was extracted and transformed into *E. coli* Transetta (DE3) (TransGen Biotech). A positive transformant was grown in LB medium, shaken at 220 rpm at 37°C, and protein expression was induced with the addition of 0.5 mM isopropyl-β-d-thiogalactopyranoside overnight at 18°C after the culture had grown to an OD_600_ of 0.5. Bacteria were harvested and lysed *via* ultrasonication, and Ni-NTA affinity chromatography was used to purify recombinant cathepsin L (rCathepsin L) with the His tag, followed by desalting via extensive dialysis against PBS (pH 7.4).

### Preparation of Polyclonal Antibodies against rCathepsin L

rCathepsin L (50 µg) was emulsified in 50 µL of complete Freund’s adjuvant (Sigma, St. Louis, MO, USA) and injected into mice of 6 and 8 weeks of age *via* multipoint subcutaneous implantation. Second and third inoculations with Freund’s incomplete adjuvant (Sigma, St. Louis, MO, USA) were performed on day 16 and day 30, while the fourth inoculation was performed on day 37 without any adjuvant. Serum was collected from blood samples 1 week after the fourth inoculation. A polyclonal IgG against rCathepsin L was prepared using a protein A column (GE Healthcare, Sweden), as described previously (28).

### Cathepsin L Western Blotting

Hemocytes were collected and lysed in RIPA buffer (50 mM Tris, pH 7.4, 150 mM NaCl, 1% NP-40, 0.5% sodium deoxycholate, 0.1% SDS) supplemented with complete protease inhibitor cocktail (Roche). Lysates were centrifuged at 12,000 × *g* for 10 min following incubation on ice for 15 min. The supernatant was separated using SDS-PAGE and electroblotted from the gel onto a nitrocellulose membrane. The membrane was incubated with 5% BSA in TBS-Tween (25 mM Tris–HCl, pH 7.8, 190 mM NaCl, 0.1% Tween-20) at room temperature for 1 h followed by incubation with polyclonal IgG against anti-rCathepsin L (1:2,000-diluted in PBS-Tween containing 3% BSA) at 4°C overnight. The membrane was extensively washed by PBS-Tween and further incubated with goat anti-mouse IgG conjugated with HRP (1:5,000-diluted in PBS-Tween) at room temperature for 1 h. After extensive washing, the immune-blotted protein bands on this membrane were visualized using an enhanced HRP-DAB chromogenic substrate kit (Tiangen) according to the manufacturer’s instruction.

### Flow Cytometric Analysis

Hemocytes were incubated with phycoerythrin (PE)-labeled latex beads (2 µm) at 1:100 (hemocytes: beads) ratio with continuous rotation (30 rpm) at 18°C for 3 h. The cells were fixed using 4% paraformaldehyde (PFA) for 10 min followed by permeabilization in 0.1% Triton X-100 for 10 min. Cells were blocked with 3% BSA in PBS-Tween for 1 h, and incubated with polyclonal IgG against rCathepsin L (1:100-diluted in PBS-Tween) at room temperature for 1 h. Hemocytes were extensively washed by PBS-Tween, incubated with FITC-labeled secondary antibodies (1:200-diluted in PBS-Tween) for 1 h, and analyzed using a FACS Aria II flow cytometer (Becton Dickinson Biosciences). The expression of cathepsin L in phagocytes (PE-positive) and non-phagocytic cells (PE-negative) was determined using the BD FACS DIVA software (Becton Dickinson Biosciences). The experiment was performed three times.

### FITC-Labeling of Microbes

Cultures of the bacterial species, *V. splendidus*, were grown in 2216E media at 28°C and shaken at 220 rpm for 12 h. The cultured bacteria were collected *via* centrifugation at 6,000 rpm for 15 min, washed three times with PBS (pH 7.4), and fixed for 15 min in 4% PFA at room temperature. Microbes were washed twice with 0.1 M NaHCO_3_ (pH 9.0) and mixed with FITC (1 mg/mL; Sigma, USA) in 0.1 M NaHCO_3_ (pH 9.0) at room temperature overnight with continuous gentle stirring. Afterward, the FITC-labeled microbes were washed again three times with PBS (pH 7.4).

### Confocal Microscopy

The confocal microscopy protocols used in this study were performed as previously described (28). Hemocytes were plated onto glass-bottom culture dishes and incubated with FITC-labeled *V. splendidus* at a 1:100 (hemocytes: bacteria) ratio for 1 h. Cells were washed with PBS and fixed with 4% PFA at room temperature for 15 min before they were permeabilized with 0.1% Triton X-100 for 10 min. Cells were blocked with 3% BSA in PBS-Tween for 1 h, and incubated with polyclonal IgG against rCathepsin L (1:100-diluted in PBS-Tween) at room temperature for 1 h. Hemocytes were extensively washed by PBS-Tween, incubated with Alexa Fluor 594-labeled goat anti-mouse IgG (1:500-diluted in PBS-Tween) for 1 h. This was followed by incubation in 4′, 6-diamidino-2-phenylindole (US Everbright, Inc.) for 10 min. The hemocytes were washed and fluorescence images were captured using a Carl Zeiss LSM 710 confocal microscope (Carl Zeiss, Germany).

### Statistical Analysis

A two-sample Student’s *t* test was used for all comparisons between groups in this study. Statistical analyses were carried out using the GraphPad Prism 5 software, and all results are reported as means ± SEM. Statistical significance was defined at *p* ≤ 0.05.

## Results

### Phagocyte Efficiently Sorting for Multi-Omics

A general lack of phagocyte-specific antibodies and cell lines has greatly hindered previous functional studies of these cells in bivalve mollusks. Thus, to obtain comprehensive insights into phagocytic killing in the Pacific oyster, *C. gigas*, an alternative strategy based on the phagocytosis of FITC-labeled latex beads was used to differentiate these cells from non-phagocytic hemocytes *via* FACS. Flow cytometry separated the phagocytic and non-phagocytic hemocytes with a high level of purity (95.2 and 98.6%, respectively) (30, 34). A total of 2.9 × 10^7^ phagocytic and 8.6 × 10^7^ non-phagocytic hemocytes were sorted efficiently, and high-throughput sequencing and iTRAQ coupled with LC-ESI-MS/MS enabled the identification of DEGs and DEPs between phagocytic and non-phagocytic hemocytes by both bioinformatics analysis plus validation at mRNA and protein levels (Figure 1).

### Transcriptomic Analysis of DEGs in Phagocytes

**Figure 2:**
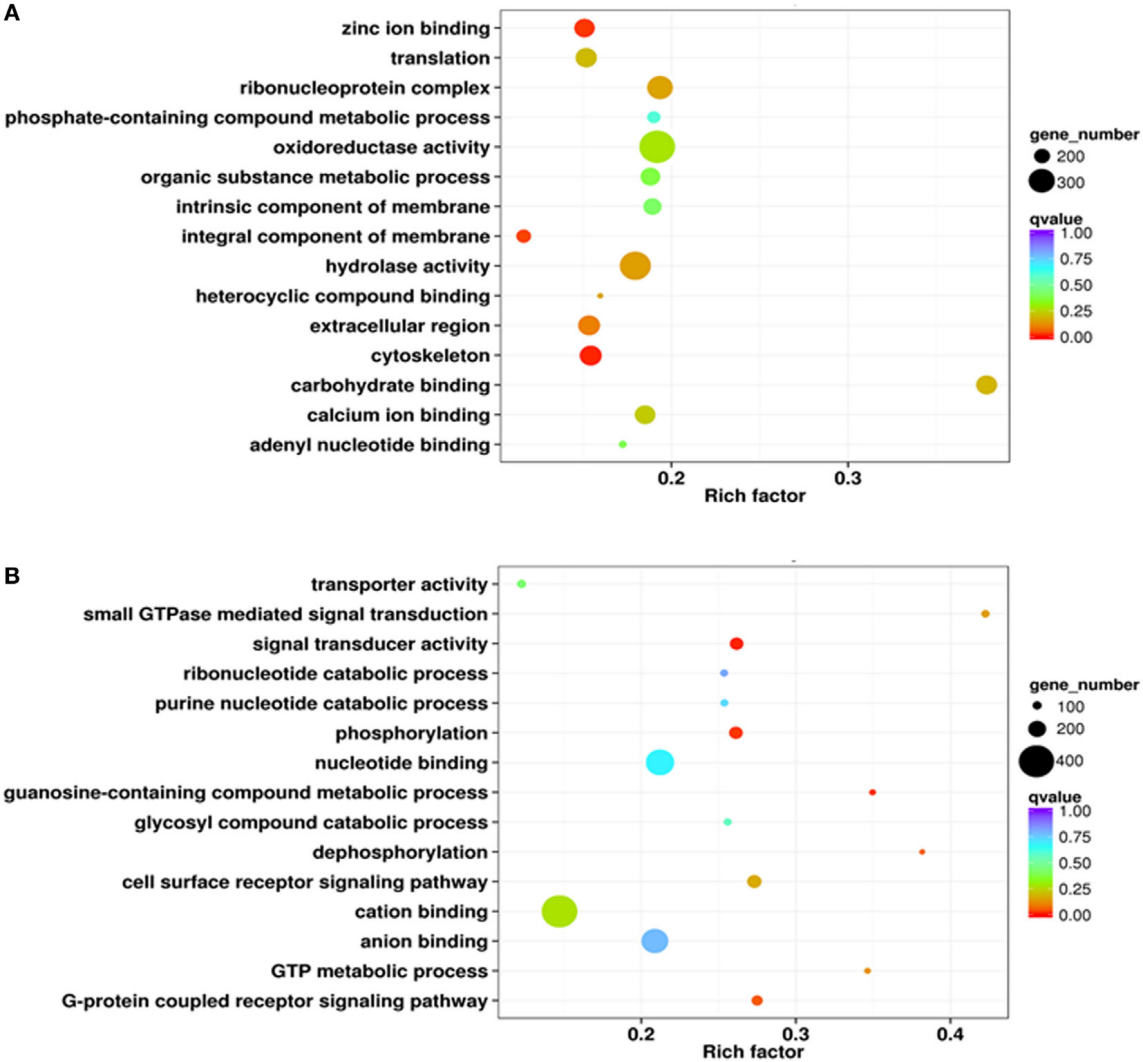
Gene ontology enrichment analysis of differentially expressed genes (DEGs) from the transcriptome. Enrichment analysis results are presented here in the form of scatterplots of high **(A)** and low expressed **(B)** phagocyte DEGs. The enrichment factor is the ratio between the DEG number and the number of all genes in a certain gene enrichment term. The sizes of the dots on these plots denote the number of DEGs, while colors correspond to the *q* value range.

A series of phagocytic and non-phagocytic hemocyte transcriptome libraries were constructed and sequenced using the SOLID4 high-throughput RNA-seq platform. Low-quality sequence regions and adapter sequences were removed and clean reads were mapped onto the *C. gigas* genome. This enabled entire sets of unigenes from both phagocytic and non-phagocytic hemocytes to be identified and annotated. Results reveal a total of 4,992 significantly high expressed genes and 3,524 significantly low expressed genes in phagocytic hemocytes relative to non-phagocytic hemocytes. The significantly high expressed genes were assigned to three GO categories: cellular components, molecular functions, and biological processes. Results indicated that the most abundant high expressed genes in phagocytes encoded integral membrane components (392 genes), while most of the others encoded nuclear proteins (86 genes), ribosomal proteins (74 genes), and microtubules (36 genes) (Figure S1A in Supplementary Material). The molecular functional categories encoded by the high expressed genes were dominated by molecular binding to zinc ions (355 genes), ATP (181 genes), calcium ions (169 genes), and carbohydrates (110 genes), as well as enzymatic activity, including oxidoreductase (98 genes), ligase (64 genes), monooxygenase (54 genes), and hydrolases (48 genes) (Figure S1B in Supplementary Material). A large number of genes within the GO category of biological processes were classified in this analysis as related to oxidation–reduction activity (249 genes), while others were assigned to stress responses (85 genes), translation (84 genes), and G-protein coupled receptor signaling pathways (74 genes) (Figure S1C in Supplementary Material). GO term enrichment analysis revealed a number of DEG molecular characteristics within phagocytic hemocytes. A scatterplot of these data revealed that a higher proportion of high expressed DEGs encoded membrane components (409 genes), oxidoreductase (392 genes), and hydrolase activity (347 genes), as well as the ribonucleoprotein complex (294 genes) (Figure 2A).

Results show that significantly low expressed genes categorized as cellular components mainly encoded proteins located in the extracellular region (35 genes) plus some with functions in the transferase complex (21 genes) (Figure S1D in Supplementary Material). The molecular functional categories of these genes were mainly attributable to molecular binding with ATP (194 genes), zinc (152 genes), and calcium ions (85 genes), as well as G-protein coupled receptors (107 genes) (Figure S1E in Supplementary Material). A number of low expressed genes within the biological processes GO category were assigned to oxidation–reduction processes (137 genes), the G-protein coupled receptor signaling pathway (124 genes), and transcription regulation (85 genes) (Figure S1F in Supplementary Material). Enrichment analysis also suggested that a large number of low expressed DEGs encoded proteins with functions in binding cations (404 genes), nt (321 genes), and anions (302 genes) (Figure 2B).

### iTRAQ Analysis of Phagocyte DEPs

**Figure 3:**
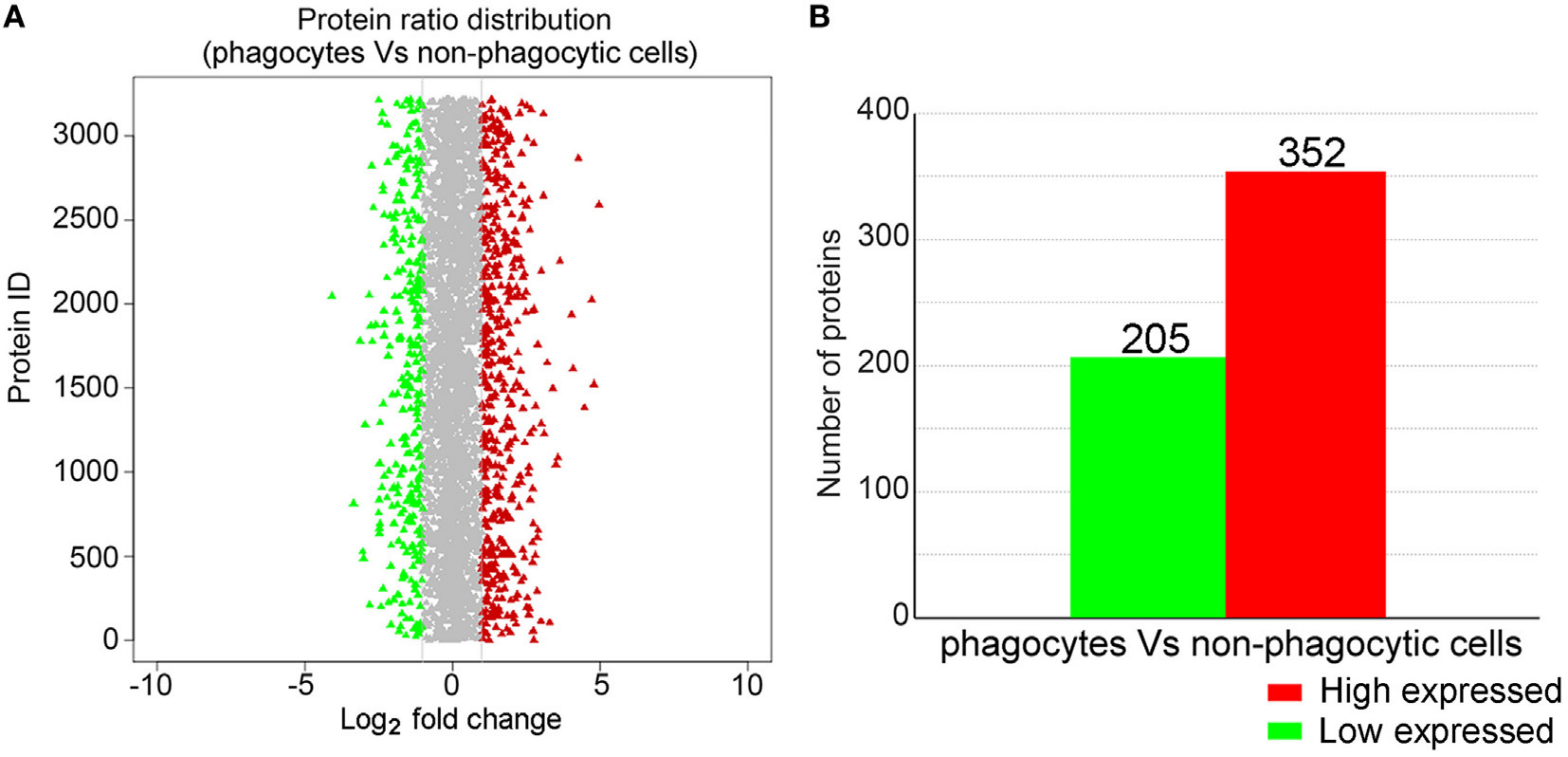
iTRAQ analysis of proteins differentially expressed in phagocytes. **(A)** This chart illustrates the overall protein expression level identified in phagocytes. The average log_2_-fold change for each protein is plotted, while those identified as either significantly high or low expressed are highlighted in red and green, respectively. **(B)** Statistical analysis of high and low expressed phagocyte differentially expressed proteins.

**Figure 4:**
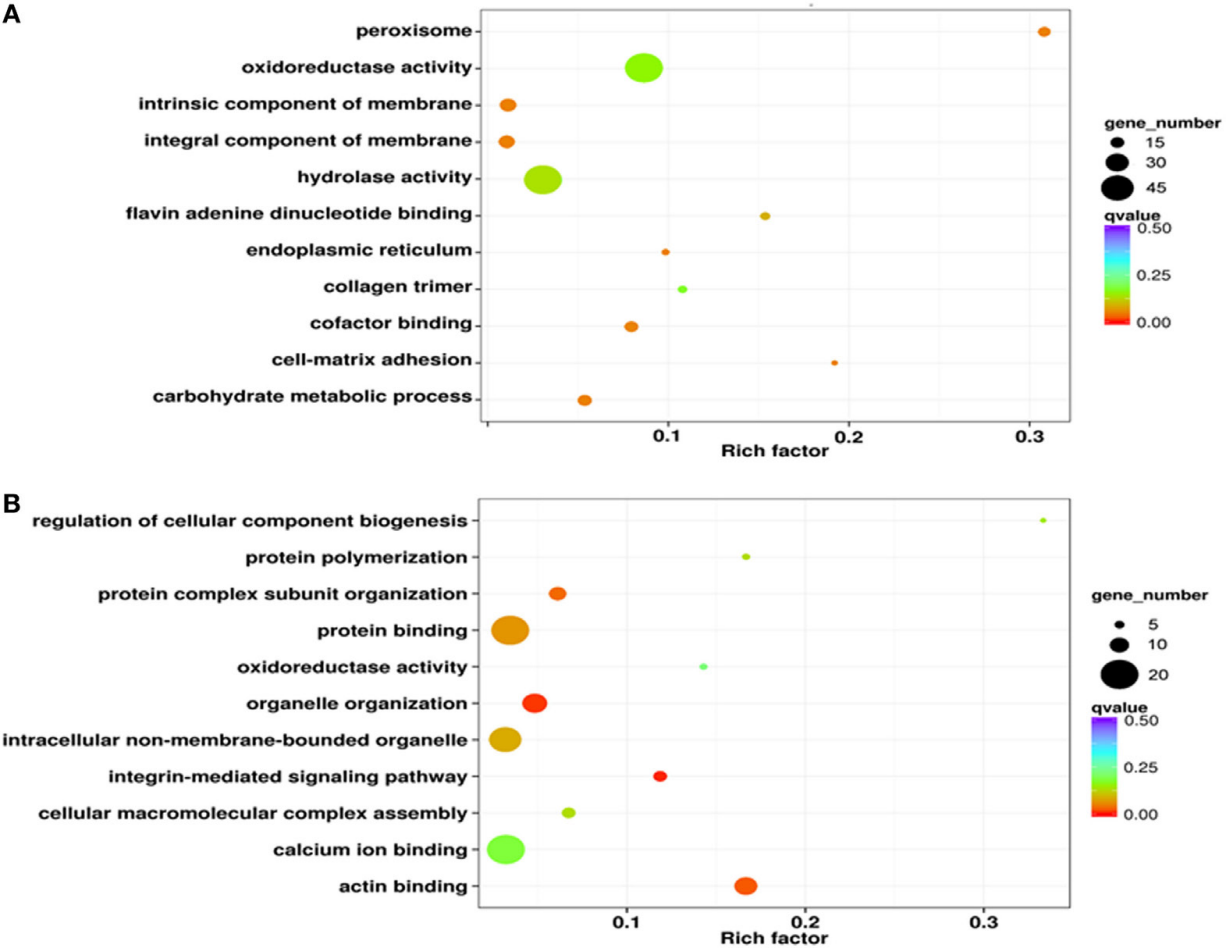
Gene ontology enrichment analysis of differentially expressed proteins from iTRAQ. The results of this enrichment analysis are presented as scatterplots of high **(A)** and low expressed **(B)** phagocyte differentially expressed proteins (DEPs). The enrichment factor in this case is the ratio of the DEP number to the number of all proteins in a certain enrichment term; the dot size in this figure denotes the number of DEPs, while colors correspond to the *q* value range.

iTRAQ analysis was performed to further determine phagocyte DEPs (Figure 3A). Data showed that compared with proteins expressed in non-phagocytic hemocytes, 352 were high expressed in phagocytes, while 205 were low expressed (Figure 3B). These DEPs were, therefore, annotated and the high expressed DEPs were assigned to one of three GO categories. For results categorized as cellular components, the most abundantly high expressed phagocyte proteins were integral membrane components (25 proteins), were associated with the extracellular region (12 proteins), or were collagen trimers (11 proteins) (Figure S2A in Supplementary Material). Components of the molecular function category were mainly comprised of hydrolases (30 proteins), oxidoreductases (22 proteins), and calcium ion-binding proteins (18 proteins) (Figure S2B in Supplementary Material), while a large number within the biological processes GO category were assigned to oxidation–reduction (70 proteins) and proteolysis (21 proteins) (Figure S2C in Supplementary Material). The GO term enrichment analysis scatterplot shows that high expressed DEPs were dominated by those associated with oxidoreductase (71 proteins) and hydrolase activities (66 proteins) (Figure 4A).

The results of this analysis showed that low expressed proteins in phagocytes were mainly classified within either the protein complex (17 proteins) or were integral membrane components (15 proteins) (Figure S2D in Supplementary Material). The molecular functional categories of these significantly low expressed proteins were mostly proteins with binding functions for calcium (20 proteins) or ATP (15 proteins) (Figure S2E in Supplementary Material). Indeed, a large proportion of the low expressed proteins were likely involved in biological processes including oxidation-reduction (21 proteins) and cell adhesion (10 proteins) (Figure S2F in Supplementary Material). GO enrichment analysis results revealed that a number of low expressed DEGs had functions for binding calcium ions (20 proteins) and other proteins (20 proteins) as well as intracellular non-membrane-bound organelles (17 proteins) (Figure 4B).

### The DEP Pathway Network Within Phagocytes

**Figure 5:**
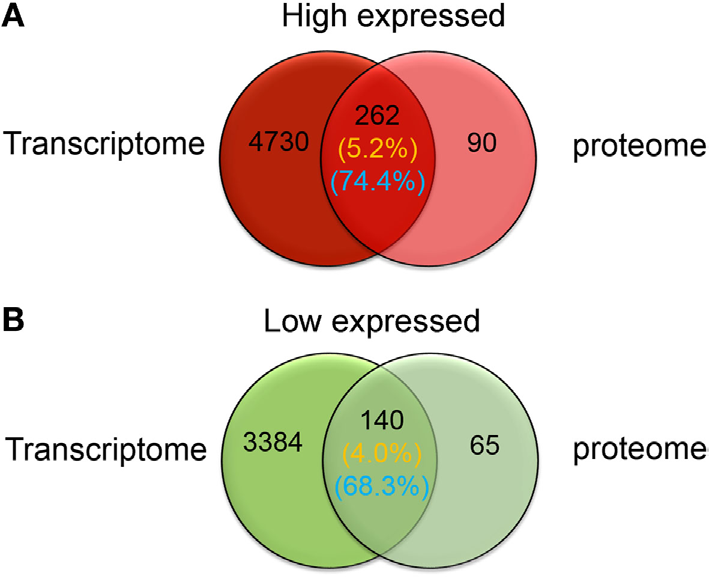
Venn-regional analysis of phagocyte differentially expressed genes and differentially expressed proteins. These diagrams illustrate overlap between significantly high (red) and low expressed (green) genes and proteins. Transcriptomic and quantitative proteomic analyses reveal a total of 4,992 genes and 352 proteins that are significantly high expressed in phagocytes **(A)**, and 3,524 and 205 that are significantly low expressed, respectively **(B)**. The percentages of overlapping high and low expressed molecules at messenger RNA and protein levels are indicated on this figure and are labeled with yellow and blue colors, respectively.

The sets of significantly high and low expressed genes and proteins identified *via* transcriptome and proteome analyses were compared to confirm phagocyte differential expression patterns. Results showed that of the 352 significantly high expressed proteins identified within the phagocyte proteome, expression of 262 corresponding genes were also significantly high expressed (Figure 5A), which was approximately 74.4% concordance between these two data sets. Furthermore, 140 of the 205 significantly low expressed proteins identified within the proteome corresponded with transcriptionally low expressed genes (Figure 5B), which was approximately 68.3% concordance. Thus, both transcriptomic and quantitative proteomic analyses suggested that protein translation was tightly coupled to gene transcription in *C. gigas* phagocytes.

**Figure 6:**
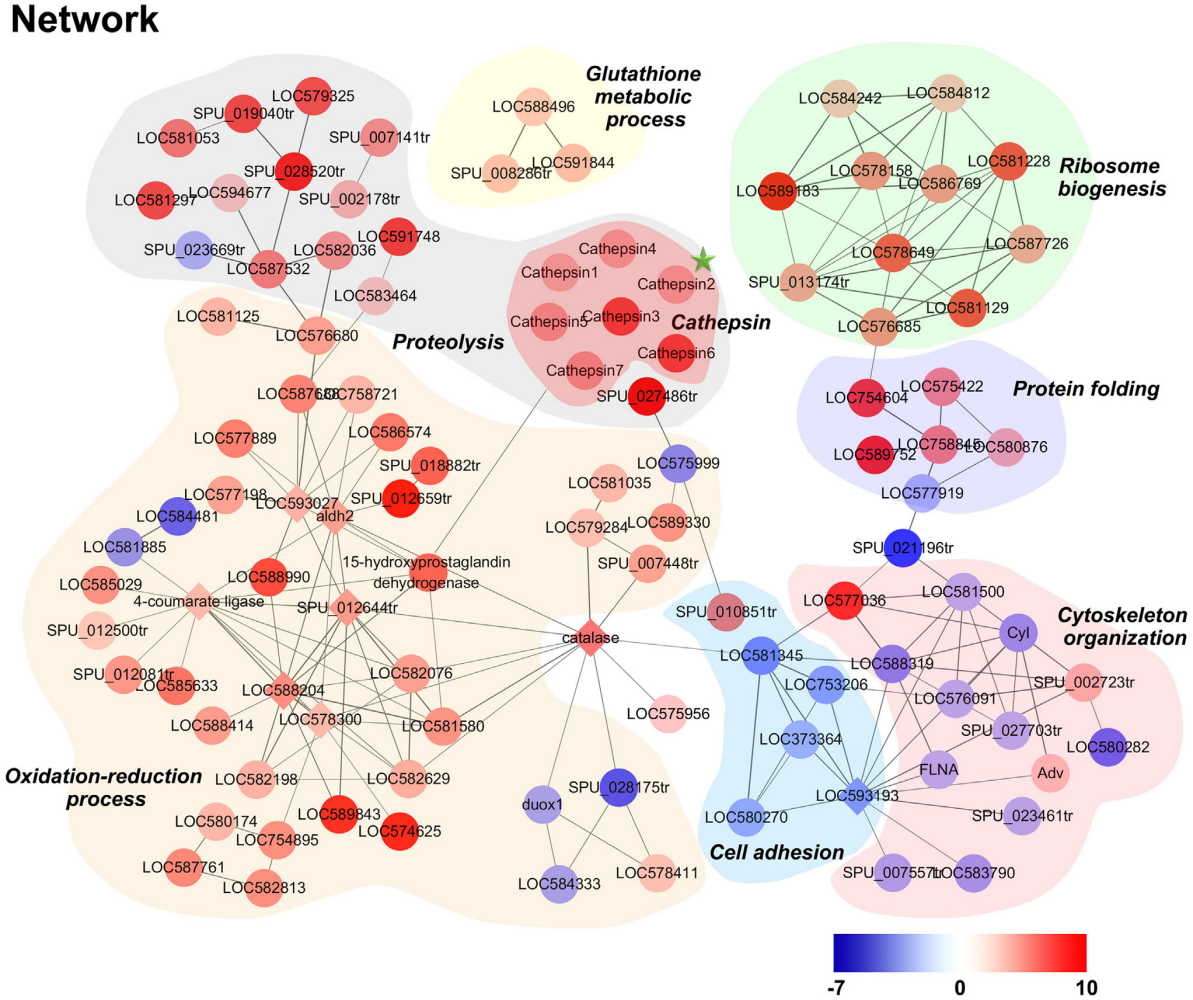
Modulation network of differentially expressed proteins (DEPs) in phagocytes. DEPs were analyzed using the software STRING and imported into Cytoscape for manual curation and grouping. The node colors on this figure correspond to absolute maximum log_2_ ratio-of-ratios; significant increases and decreases in phagocyte DEPs are highlighted in red and blue, respectively.

A DEP-based network was constructed using interactions from the STRING database to further elucidate the molecular features of killing in phagocytes. When dominant proteins were grouped and denoted with different colors (Figure 6), results highlighted a number of significantly high expressed proteins involved in oxidation-reduction and proteolysis processes in phagocytes. Notably, seven lysosomal protease cathepsin L isoforms were all high expressed in phagocyte proteolysis processes, while glutathione metabolism, ribosome biogenesis, and protein folding-related processes were all also high expressed in phagocytes.

### qPCR Analysis of Selected Genes in Phagocytes and Non-Phagocytic Hemocytes

**Figure 7:**
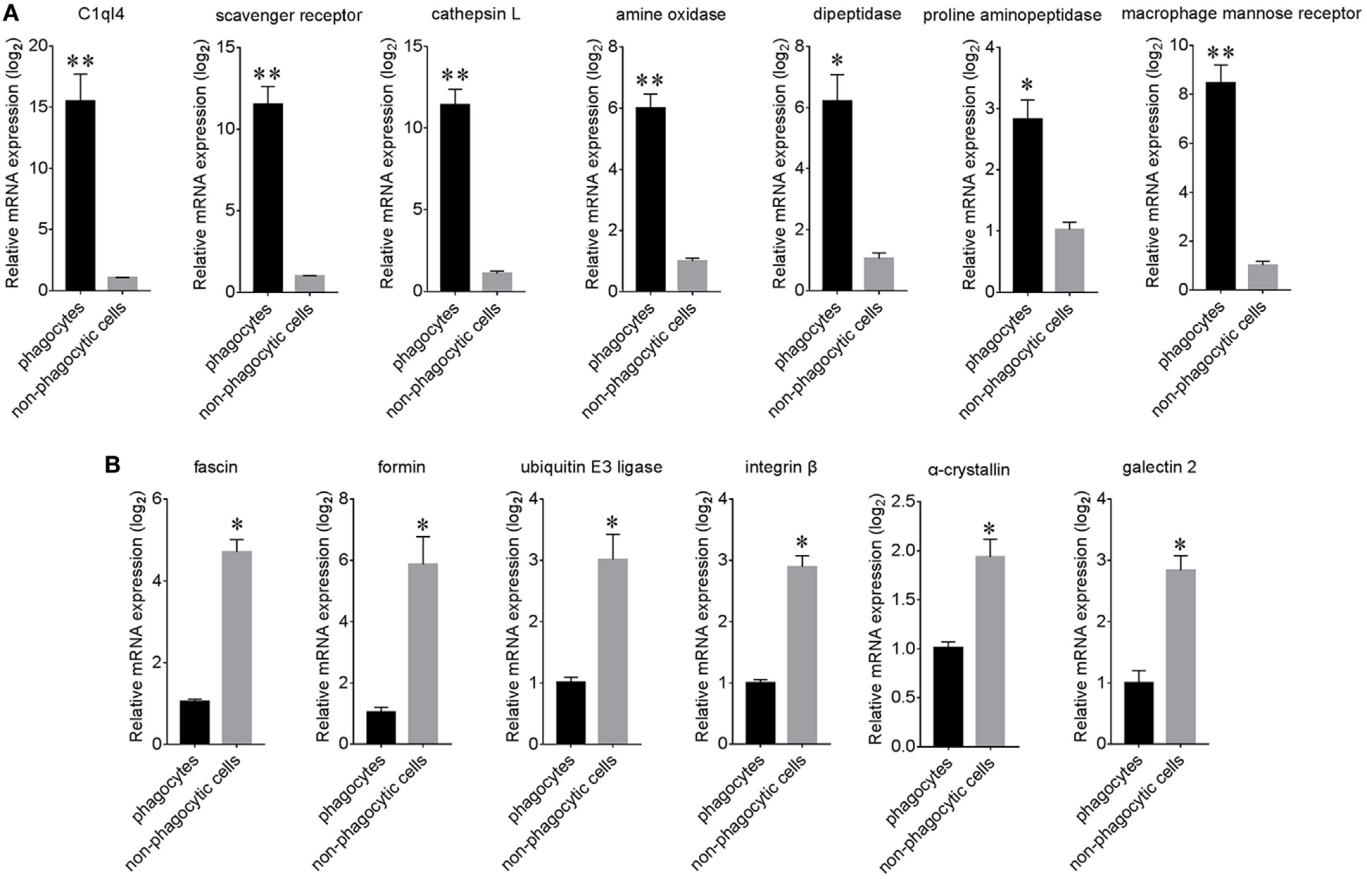
Relative expression analysis of differentially expressed proteins (DEPs) using qPCR. The expression of significantly high expressed **(A)** and low expressed **(B)** proteins within phagocytes and non-phagocytic hemocytes was determined using qPCR. Results are indicated here at the messenger RNA level for each phagocyte gene in relation to their level in non-phagocytic hemocytes (*n* = 6), *p < 0.05, **p < 0.01.

A total of 13 candidate proteins, including seven high expressed and six low expressed examples in phagocytes, were selected for mRNA expression verification by qPCR. Results showed that C1q-like protein 4 (C1ql4), a scavenger receptor (SR), a cathepsin L (CGI_10003564, isoform 2 that labeled with green asterisk in Figure 6), amino oxidase, dipeptidase, proline aminopeptidase, and macrophage mannose receptor (MMR) were all significantly high expressed in phagocytes (Figure 7A), while fascin, formin, ubiquitin E3 ligase, integrin β, α-crystallin, and galectin 2 were all significantly low expressed (Figure 7B). The differential expression of these proteins, as revealed by iTRAQ, is summarized in Table S7 in Supplementary Material. These DEGs and DEPs in phagocytes indicated a functional differentiation in oyster hemocytes.

### Significant Expression of Cathepsin L Protein in Phagocytes

**Figure 8:**
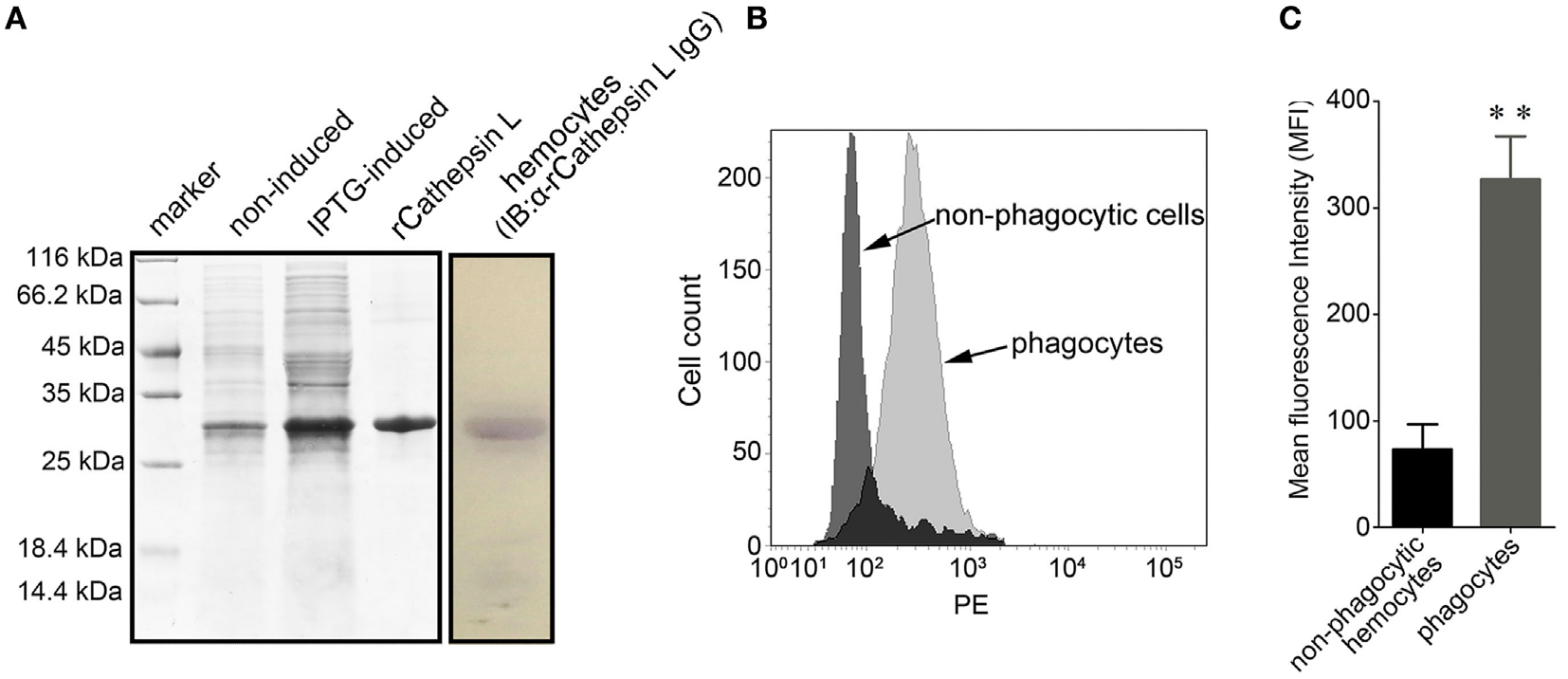
Differential expression of cathepsin L in hemocytes. **(A)** In this case, rCathepsin L was expressed and the purified form was separated using SDS-PAGE followed by Coomassie brilliant blue staining (left box). Western blotting analysis was then performed using polyclonal IgG against rCathepsin L (right box). IB: immunoblot. **(B)** Histogram to show the relative expression level of cathepsin L in phagocytes (gray) and non-phagocytic hemocytes (black) determined by flow cytometry. **(C)** The mean fluorescence intensity of cathepsin L was statistically calculated; these results are expressed as means ± SEM (*n* = 5), **p < 0.01.

The coding sequence of cathepsin L (CGI_10003564, isoform 2 that labeled with green asterisk in Figure 6) was cloned and the corresponding recombinant protein (rCathepsin L) was purified *via* Ni-NTA affinity chromatography. SDS-PAGE analysis revealed the presence of a single protein band at about 27 kDa (Figure 8A). A polyclonal antibody against rCathepsin L was prepared and western blotting analysis revealed a distinct single immune-precipitated band with a similar molecular weight predicted by the target sequence. This result suggested a high binding specificity of the polyclonal antibody against cathepsin L (Figure 8A; Figure S3 in Supplementary Material). The cathepsin L expression level in hemocytes represented by mean fluorescence intensity (MFI) was, therefore, determined using flow cytometry (Figure 8B) and results showed that the expression level of this protein in phagocytes (MFI: 327 ± 51) was significantly higher (*p* < 0.01) compared to non-phagocytic hemocytes (MFI: 83 ± 26) (Figure 8C).

### Differentially Expressed Cathepsin L Colocalized With the Engulfed Bacterial Pathogen

**Figure 9:**
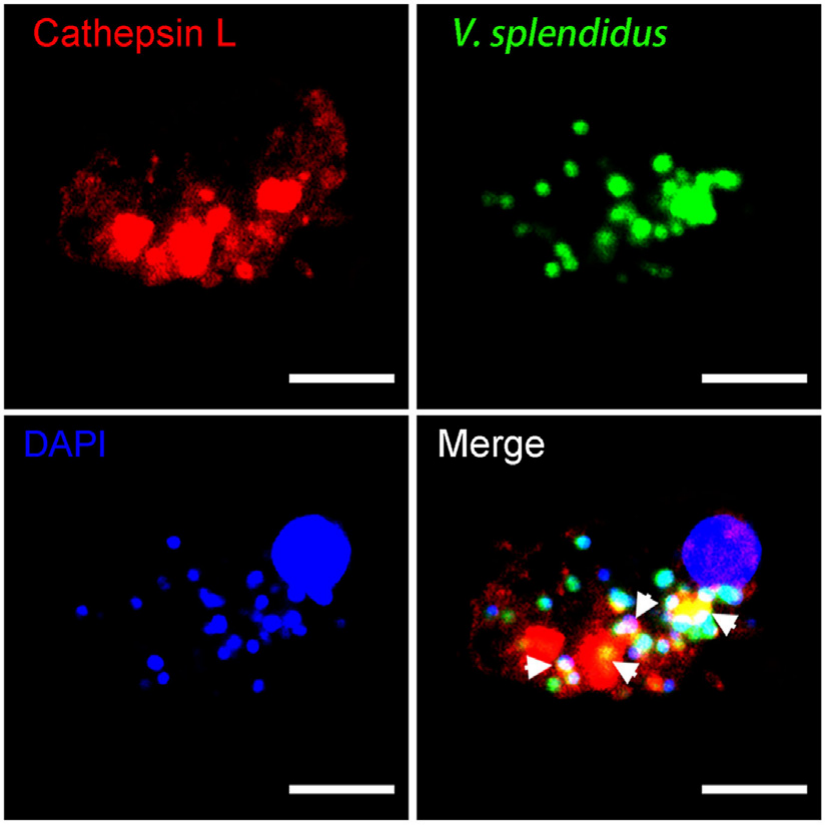
The involvement of cathepsin L in hemocyte phagocytosis of *Vibrio splendidus*. Hemocytes were incubated with FITC-labeled *V. splendidus* to allow hemocyte phagocytosis. Subsequent to cell fixation and permeabilization, hemocytes were stained with polyclonal IgG against rCathepsin L and then subjected to Alexa Fluor 594-labeled anti-mouse IgG antibody staining. diamidino-2-phenylindole (DAPI) was used to mark the cell nucleus, and each image was captured at a single focal plane using confocal microscopy. Interactions between cathepsin L and engulfed microbes are denoted here with white arrows; scale bar: 10 µm.

The immunological role of cathepsin L during phagocytosis was investigated using confocal microscopy. The image reveals that this protein was localized in subcellular compartments with an uneven distribution throughout the cytoplasm. A number of bacteria were engulfed during oyster hemocyte phagocytosis toward *V. splendidus*, and this pathogen was colocalized with cathepsin L (Figure 9). These findings suggested that the bacteria were enclosed within phagolysosomes during hemocyte phagocytosis, and that lysosomal cathepsin L may have been functioning in both microbicidal and proteolytic activities.

## Discussion

Phagocytes have been shown to play pivotal roles in the innate immune defense responses of both vertebrates and invertebrates (35–37). Neutrophils in mammals, for example, exhibit antimicrobial activity toward both bacteria and fungi *via* ROS upregulation (38, 39). Furthermore, macrophages produce a large variety of cytokines, including tumor necrosis factor-α, interferon-β, and interleukin-6 following pathogen engulfment, and these cytokines activate both innate and adaptive immune responses to bacterial infection (40, 41). Previous research has also shown that the phagocytosis of enterohemorrhagic *E. coli* induces inflammasome activation in bone marrow-derived macrophages in mice (42). Although the molecular mechanisms underlying phagocytosis as part of the innate immune response have been extensively studied in mammals, hemocyte heterogeneity combined with a lack of phagocytic lines and specific cell markers have greatly impeded research in this area in invertebrates. Flow cytometry combined with multi-omics analyses were, therefore, undertaken to obtain more comprehensive insights into the molecular mechanisms of phagocytosis in an economically important marine invertebrate, the oyster *C. gigas*. Here, a series of FITC-labeled latex beads were used as engulfment targets for hemocytic phagocytes, which enabled phagocytes to be marked with strong, clear fluorescent signals for FACS analysis. As latex beads are inert polystyrene particles, their use also avoids the unnecessary stimulation of oyster hemocytes during phagocytosis (43, 44). Transcriptome and iTRAQ quantitative proteome analyses were subsequently performed for global identification of both DEGs and DEPs in phagocytes, respectively. The strategy proposed here represents an important step forward in the study of phagocytosis in invertebrates, previously impeded due to a lack of appropriate cell lines and phagocytic-specific antibodies.

The phagocytes of *C. gigas* exhibit a number of significant differences in their morphological characteristics (30) as well as their molecular components and related signaling pathways compared to the non-phagocytic hemocytes of this species. The transcriptomic and quantitative proteomic analyses presented here reveal that a large proportion of high expressed genes and proteins may be membrane-associated within phagocytes, while their low expressed counterparts are mainly concentrated within components of protein complexes. Proteome and qPCR analytical results show that membrane receptors, including SR and MMR, are high expressed in phagocytes, and SR is an important transmembrane immune receptor involved in pathogen recognition and clearance (45). Previous work has shown that over 200 genes that encode more than 1,000 SR cysteine rich domains in the purple sea urchin *Strongylocentrotus purpuratus*, which exhibits a 10-fold expansion relative to vertebrates (46, 47). Genes encoding SR are also highly expressed in the American oyster *Crassostrea virginica* in response to bacterial challenges (48). Furthermore, a novel SR type is present in the scallop *Chlamys farreri* that is significantly upregulated following lipopolysaccharide, peptidoglycan, and β-glucan stimulation (49). In contrast, MMR is a type I membrane-bound immune receptor that is highly expressed on human macrophages, and modulates phagocytosis during pathogen infections (50, 51). Previous research in this area has shown that MMR is also highly expressed in the hemocytes of the scallop *Pecten maximus* where it is involved in the regulation of immune response (52) and is the target of microRNAs associated with the neural-endocrine-immune system (53). A large number of high expressed phagocyte proteins were identified in this study that are involved in oxidative metabolism and proteolysis. Oxidative metabolism is important for immune defense as hemocytes from *P. maximus* and *C. gigas* initiate an oxidative burst during phagocytosis when exposed to different bacterial strains (54). Proteolytic processes, especially endo- and lysosomal proteolysis, are crucial for the innate immune responses of both invertebrates and vertebrates (55). Indeed, previous work has suggested that the phagolysosome is involved in the destruction of the parasite *Marteilia sydneyi* by hemocytes of the Sydney Rock oyster *Saccostrea glomerata* (56).

The lysosome is essential for pathogen–host interactions within which engulfed microbes are killed and degraded (57, 58). The results of both the transcriptomic and proteomic analyses presented here for the Pacific oyster *C. gigas* show that the genes and proteins involved in the lysosomal proteolytic pathway are significantly high expressed in phagocytes. This suggests that phagocytes may have a higher lysosomal proteolytic capacity to overcome harsh and dynamically changing microbial challenges. In addition, cathepsin is one of the most important proteases within the lysosome-mediated antimicrobial pathway because it is involved in the complete disintegration of large complex structures including phagocytosed microbes (59). Notably, seven cathepsin L proteins were identified within phagocytes among proteolysis-related compounds, suggesting that cathepsin-mediated lysosomal proteolysis may be strongly active during *C. gigas* hemocyte phagocytosis. Furthermore, cathepsin is associated with bactericidal activity against infections in both vertebrates and invertebrates: cathepsin L in human macrophages enhances the proteolytic activity against phagocytosed *Salmonella typhimurium* (60). Moreover, cathepsin D exhibits antimicrobial action toward *Listeria monocytogenes* in both fibroblasts and macrophages of mice (61). Research in lower invertebrates has also shown that cathepsin L within the digestive glands of the pearl oyster *Pinctada fucata* is significantly up-regulated following stimulation with *Vibrio alginolyticus* (62), while cathepsin L in the hemocytes of the Hawaiian bobtail squid *E. scolopes* is involved in the modulation of the beneficial luminescent bacterium *Vibrio fischeri* (63). Previous work has shown an increase in the expression of cathepsin L following infections of the *Macrobrachium rosenbergii* nodovirus and white spot syndrome virus in the freshwater prawn *M. rosenbergii* (64), while colocalization of cathepsin L with phagocytosed *V. splendidus* in the phagolysosome suggests that this protein probably has direct antimicrobial activity during pathogen phagocytosis as a component of the *C. gigas* innate immune response.

Oxidative killing is one of the most critical immune responses that contributes to pathogen resistance (65, 66). Previous research has shown that *C. gigas* granulocytes together with hyalinocytes significantly increase the production of oxidative metabolism products after the phagocytosis toward zymosan particles, and this oxidative metabolism plays an important role in the interaction between oysters and pathogenic vibrios (67). The reactions involved in oxidative process produce superoxide following the microbial stimulation of hemocytes from both the eastern oyster *C. virginica* and the mussel *Geukensia demissa* (68), while the production of ROS in hemocytes of the mussel *Mytilus galloprovincialis* may also be induced by yeast phagocytosis (69). The transcriptome and proteome analyses presented here both show that a large number of proteins, including amino, peroxisomal acyl-coenzyme A, and d-aspartate oxidases, are expressed at higher levels during oxidative biological processes in *C. gigas* phagocytes compared with the non-phagocytic hemocytes. That these enzymes are involved in peroxisome ROS production indicates that oxidative killing mediated by these chemical species may be highly effective in *C. gigas* phagocytes. Amino oxidase, which is one of the most important members of this chemical group, is highly expressed in phagocytes and these proteins also produce hydrogen peroxide, which is known to be a highly toxic molecule with important functions in the innate immune defense (70). In the rockfish *Sebastes schlegeli*, for example, amino oxidase is expressed in skin mucus and performs an antibacterial function (71), while a similar compound within the epidermal mucus of the flounder *Platichthys stellatus* also exerts bactericidal activity in response to the methicillin-resistant bacteria *Staphylococcus aureus* (72). Additional research has also shown that the oxidative process within hemocytes plays a key role in the formation of extracellular DNA traps in *C. gigas*, and that these structures operate as antimicrobial effectors during the innate immune response (73). It is believed that the disruption of oxidative killing induces bacterial pathogenesis in oysters and the intracellular pathogen *V. splendidus* LGP32, which inhibits ROS production, also attenuates antimicrobial activities that increases pathogen survival in oyster hemocytes. This contrasts with another *V. splendidus* strain, LMG20012^T^, in which ROS inhibition significantly decreases in hemocytes following oyster infection (74, 75).

The maintenance of redox homeostasis is essential for innate immune responses (76, 77). However, although oxidative killing exhibits strong antimicrobial activity, excessive chemical stress from this process can also damage cytoplasmic organelles, induce cell death, and disrupt immune system function (78, 79). As a component of the DEP pathway crosstalk network within phagocytes, the glutathione (GSH) metabolic process is significantly high activated in phagocytes. GSH is a free radical scavenger which detoxifies ROS by acting as an electron donor (80). Previous work has shown that the GSH metabolic pathway is important for the maintenance of redox homeostasis in oysters (81, 82). Hemocytes treated with GSH depletor 1-chloro-2,4-dinitrobenzene exhibit a significant reduction in intracellular GSH content, which results in a higher ROS production and lower *C. gigas* immuno-competence (83). The higher expression level of GSH metabolic process in phagocytes therefore implies the involvement of a reduction process in balancing phagocytosis-induced oxidative bursts, which have the potential to prevent hemocyte cell death as well as possible immune dysfunction induced by the excessive presence of free radicals.

The results of this study show that a number of genes and proteins are expressed at higher levels within the non-phagocytic hemocytes of *C. gigas*, especially compared with those present in phagocytes. In particular, galectin 2 is significantly high expressed in non-phagocytic hemocytes. These molecules, which are important components of the immune system, are known as β-galactoside-binding proteins and are involved in microbial recognition, cell adhesion, and migration (84, 85). One galectin with a quadruple-domain that is expressed in the bay scallop *Argopecten irradians* exhibits robust agglutinating activities to *Vibrio anguillarum, V. fluvialis*, and *Edwardsiella tarda* (86). Another prototype galectin isolated from *C. gigas* is thought to be associated with cell adhesion and tissue development (87). Integrin β is also highly expressed in the non-phagocytic hemocytes of *C. gigas*, and, similar to galectin, is an important immune protein that modulates cell migration and spreading. Integrin, for example, mediates cell adhesion and enhances spreading of *C. gigas* hemocytes (88). Another example, formin, is also significantly high expressed in non-phagocytic cells and is an intracellular protein that acts to modulate cell polarization (89). Formin occurs in significant amounts within the gills of the oyster *Crassostrea hongkongensis* in response to metal contamination and has an important role in controlling both gene expression and chromatin remodeling (90).

Here we show that FACS analysis combined with a multi-omics assay resulted in a more comprehensive understanding of phagocytosis in an economically important marine invertebrate, the Pacific oyster, *C. gigas*. Transcriptomic and quantitative proteomic analyses revealed a series of DEGs and DEPs, while the generation of a modulation network elucidated significantly high and low regulated pathways within phagocytic and non-phagocytic hemocytes. It is noteworthy that cathepsin L-mediated lysosomal proteolysis is highly activated in *C. gigas* phagocytes, and that this process may therefore be involved in innate immune responses to microbial infection. The results of this study provide several novel insights into the phagocytosis of hemocytes in *C. gigas* innate immunity.

## Ethics Statement

All experiments involving animals reported in this study were approved by the Ethics Committee of the Institute of Oceanology, Chinese Academy of Sciences.

## Author Contributions

SJ carried out phagocyte sorting, cloning, expression, and purification of recombinant proteins as well as mRNA and differentially expressed protein analyses. SJ, ZJ, and ZL performed RNA and protein extraction for transcriptome and proteome sequencing, while MW, CL, and JX carried out bioinformatics analyses of transcriptomic and quantitative proteomic data. SJ, LW, LQ, and LS designed the research and wrote the manuscript.

## Conflict of Interest Statement

The authors declare that the research was conducted in the absence of any commercial or financial relationships that could be construed as a potential conflict of interest.

## Footnotes

^1^http://www.oysterdb.com/.

^2^http://www.genome.jp/kegg/.

^3^http://www.iprox.org (Accessed: June 1, 2018).
