# Supplementary material for: Transcriptomic and Quantitative Proteomic Analyses Provide Insights Into the Phagocytic Killing of Hemocytes in the Oyster Crassostrea gigas
Source: Front Immunol. 2018 Jun 11;9:1280. doi: 10.3389/fimmu.2018.01280 (PMC6005338; doi:10.3389/fimmu.2018.01280)
Supplement: Supplementary file 7 [file Table_7.DOC]

**Table S7. The selected DEPs identified by iTRAQ in phagocytes of *C. gigas*.**

| **DEPs** | **Protein name** | ***C. gigas* database ID** | **Score** | **Coverage** | **Unique peptide** | **Log_2_ fold** |
| --- | --- | --- | --- | --- | --- | --- |
| **Up-regulated** |  |  |  |  |  |  |
|  | C1q-like protein 4 | CGI_10022091 | 137 | 17.5 | 2 | 2.475 |
|  | scavenger receptor | CGI_10007812 | 785 | 19.6 | 11 | 3.891 |
|  | cathepsin L | CGI_10003564 | 1521 | 34.2 | 9 | 6.536 |
|  | macrophage mannose receptor | CGI_10025847 | 91 | 6.6 | 5 | 6.135 |
|  | amine oxidase | CGI_10022845 | 1195 | 36.9 | 17 | 4.739 |
|  | dipeptidase | CGI_10002630 | 68 | 2.4 | 1 | 2.494 |
|  | proline aminopeptidase | CGI_10010897 | 261 | 29.8 | 9 | 2.049 |
| **Down-regulated** |  |  |  |  |  |  |
|  | fascin | CGI_10025410 | 2056 | 41.9 | 20 | 0.444 |
|  | formin | CGI_10007354 | 179 | 11.3 | 11 | 0.459 |
|  | ubiquitin E3 ligase | CGI_10010268 | 122 | 11.9 | 4 | 0.477 |
|  | integrin β | CGI_10015543 | 60 | 7.5 | 2 | 0.48 |
|  | α-crystallin | CGI_10005959 | 211 | 21.9 | 2 | 0.259 |
|  | galectin 2 | CGI_10015451 | 831 | 22.3 | 9 | 0.23 |
