# Supplementary material for: Transcriptomic and Quantitative Proteomic Analyses Provide Insights Into the Phagocytic Killing of Hemocytes in the Oyster Crassostrea gigas
Source: Front Immunol. 2018 Jun 11;9:1280. doi: 10.3389/fimmu.2018.01280 (PMC6005338; doi:10.3389/fimmu.2018.01280)
Supplement: Supplementary file 8 [file Data_Sheet_1.docx]

**Supplementary Figures**


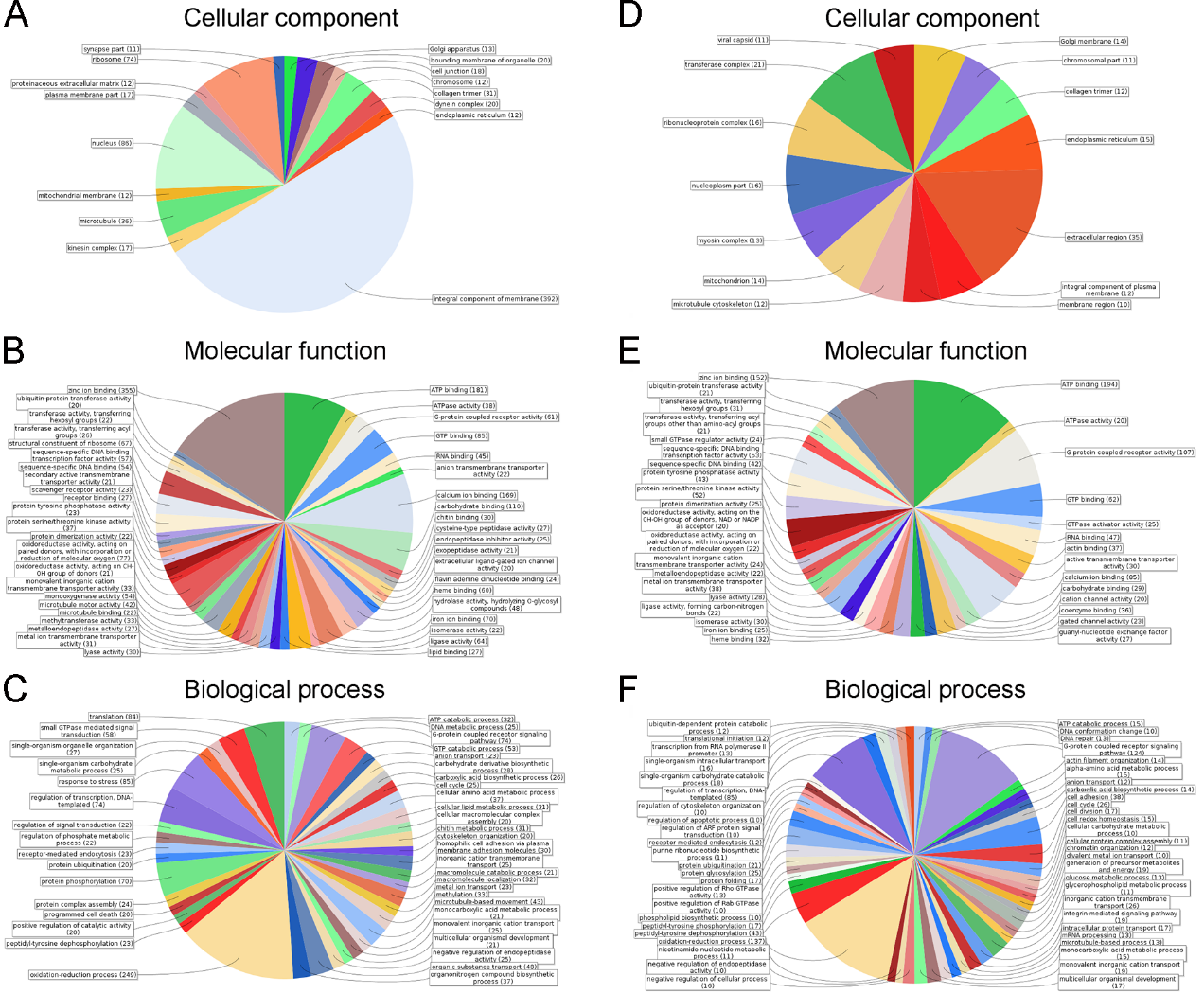


**Figure S1. Gene ontology analysis of the differentially expressed genes in phagocytes.** The distribution of significantly high expressed genes is subjected to three GO categories: **(A)** cellular component, **(B)** molecular function and **(C)** biological process. The significantly low expressed genes are categorized into **(D)** cellular component, **(E)** molecular function, as well as **(F)** biological process.


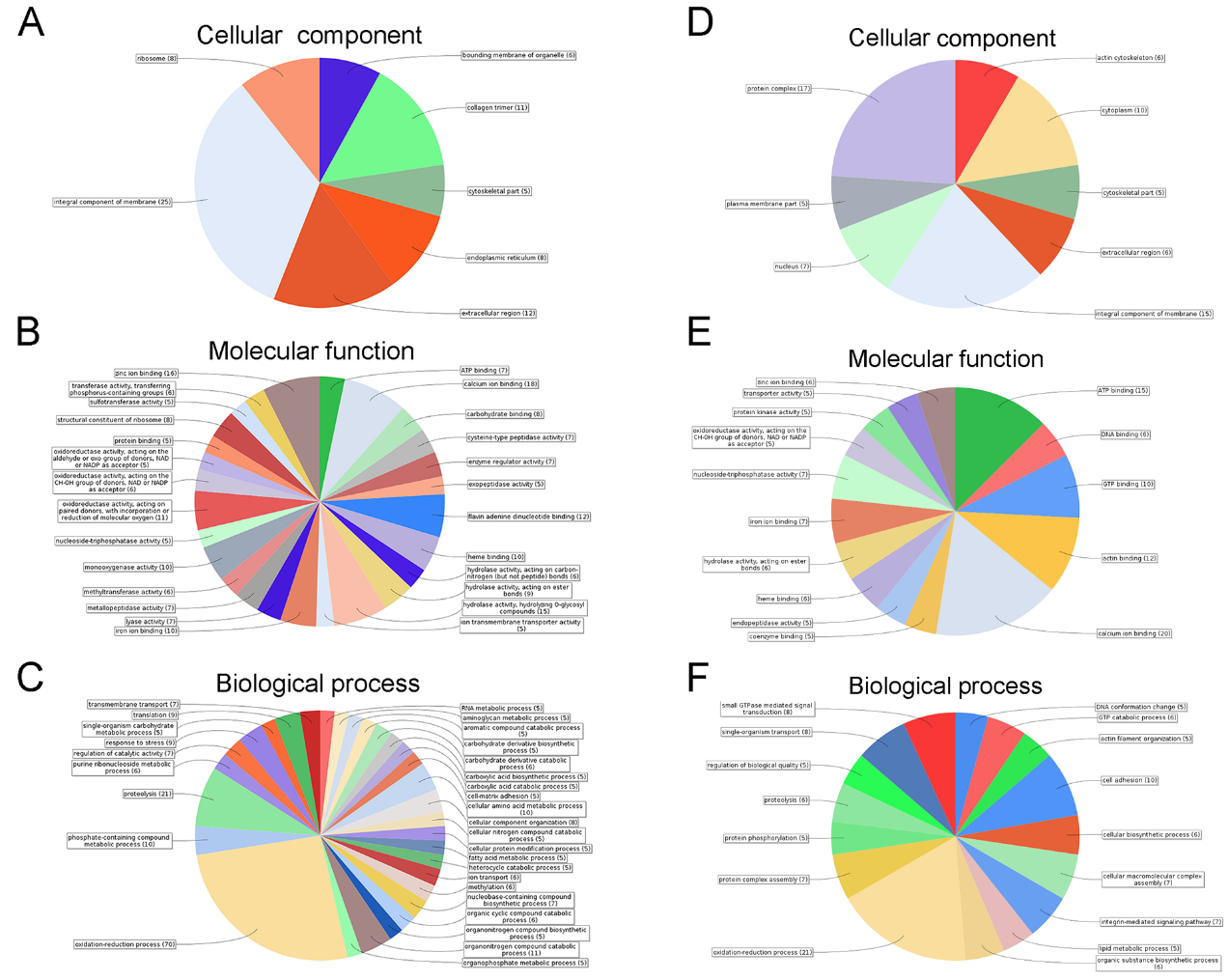


**Figure S2. Gene ontology analysis of the differentially expressed proteins in phagocytes.** The distribution of significantly high expressed proteins is subjected to three categories: **(A)** cellular component, **(B)** molecular function and **(C)** biological process. The significantly low expressed proteins are categorized into **(D)** cellular component, **(E)** molecular function as well as **(F)** biological process.


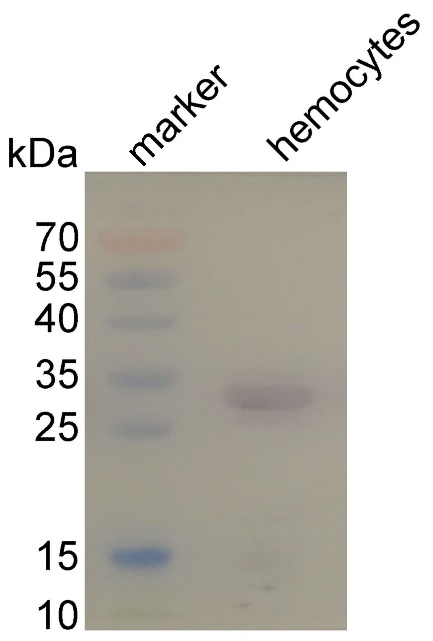


**Figure S3. The original image of Western blotting analysis of cathepsin L in Figure 8A.** Molecular weight standards are indicated by the protein ladder. Total proteins from hemocytes are transferred to the polyvinylidene fluoride (PVDF) filter and detected with the anti-rCathepsin L antibody (IgG). The cathepsin L protein band (dark blue) is revealed by using enhanced HRP-DAB chromogenic substrate.
